# Supplementary material for: Pseudomonas aeruginosa clinical blood isolates display significant phenotypic variability
Source: PLoS One. 2022 Jul 6;17(7):e0270576. doi: 10.1371/journal.pone.0270576 (PMC9258867; doi:10.1371/journal.pone.0270576)
Supplement: S1 Table — (PDF) [file pone.0270576.s003.pdf]

S1 Table. Assembly Statistics.

Assembly	RWJPA_01	RWJPA_02	RWJPA_03	RWJPA_04	RWJPA_05
Accession ID	JAIFHX000000000	JAIFHW000000000	JAIFHV000000000	JAIFHU000000000	JAMKOP000000000
# contigs (>= 0 bp)	847	1127	921	1189	1010
# contigs (>= 1000 bp)	829	1101	886	1157	910
# contigs (>= 5000 bp)	307	314	339	317	364
# contigs (>= 10000 bp)	118	102	141	99	141
# contigs (>= 25000 bp)	21	11	17	10	22
# contigs (>= 50000 bp)	2	3	2	0	2
Total length (>= 0 bp)	4852386	5245956	5447710	5229446	5627158
Total length (>= 1000 bp)	4845663	5231930	5432350	5211636	5571005
Total length (>= 5000 bp)	3407734	3233826	4009574	3124051	4142393
Total length (>= 10000 bp)	2100689	1795389	2614319	1614028	2593175
Total length (>= 25000 bp)	762570	463987	671092	334790	869438
Total length (>= 50000 bp)	112379	200839	127448	0	152390
# contigs	832	1115	897	1173	966
Largest contig	57435	95527	71860	45222	100172
Total length	4847444	5242454	5439850	5224058	5612202
GC (%)	65.32	64.77	65.39	64.83	65.06
N50	8309	6539	9183	6361	9061
N75	4405	3497	4845	3392	4773
L50	154	207	152	227	164
L75	356	482	354	511	378
# N's per 100 kbp	0	0	0	0	0

Assembly	RWJPA_06	RWJPA_07	RWJPA_08	RWJPA_09	RWJPA_10
Accession ID	JAIFHT000000000	JAIFHS000000000	JAMKOO000000000	JAIFHR000000000	JAIFHQ000000000
# contigs (>= 0 bp)	1023	1011	844	1421	1110
# contigs (>= 1000 bp)	946	951	805	1060	1073
# contigs (>= 5000 bp)	267	366	360	396	349
# contigs (>= 10000 bp)	91	152	150	148	107
# contigs (>= 25000 bp)	14	17	26	19	16
# contigs (>= 50000 bp)	2	0	1	0	1
Total length (>= 0 bp)	4678422	5619041	5473816	6104119	5443440
Total length (>= 1000 bp)	4640073	5586492	5453835	5944124	5424757
Total length (>= 5000 bp)	2880838	4017972	4251126	4292105	3587849
Total length (>= 10000 bp)	1629301	2548279	2759072	2508639	1889845
Total length (>= 25000 bp)	550028	573171	940833	661156	565430
Total length (>= 50000 bp)	175015	0	75733	0	51535
# contigs	978	983	821	1166	1087
Largest contig	109639	43214	75733	49712	51535
Total length	4663337	5609493	5465488	6017238	5435878
GC (%)	64.4	65.05	65.18	64.57	64.98
N50	6956	8775	10116	8536	7226
N75	3645	4503	5460	4379	3929
L50	175	180	148	203	206
L75	412	406	331	444	460
# N's per 100 kbp	0	0	0	0	0

Assembly	RWJPA_11	RWJPA_12	RWJPA_13	RWJPA_14	RWJPA_15
Accession ID	JAIFHP000000000	JAIFHO000000000	JAIFHN000000000	JAIFHM000000000	JAIFHL000000000
# contigs (>= 0 bp)	892	935	867	982	670
# contigs (>= 1000 bp)	865	909	829	929	648
# contigs (>= 5000 bp)	337	327	340	380	355
# contigs (>= 10000 bp)	146	130	128	145	151
# contigs (>= 25000 bp)	25	25	17	17	39
# contigs (>= 50000 bp)	4	2	1	2	11
Total length (>= 0 bp)	5513513	5371574	5042057	5737848	5530957
Total length (>= 1000 bp)	5498281	5355406	5023000	5712543	5521555
Total length (>= 5000 bp)	4096195	3816721	3703291	4239530	4683297
Total length (>= 10000 bp)	2755217	2428798	2198455	2550047	3229387
Total length (>= 25000 bp)	959343	902431	604852	628135	1500669
Total length (>= 50000 bp)	237391	118852	52085	125375	597102
# contigs	880	925	845	950	656
Largest contig	66830	62726	52085	73421	62953
Total length	5509452	5367750	5035183	5727792	5526810
GC (%)	65.21	64.53	65.26	65.14	64.74
N50	10096	8689	8758	9076	13488
N75	4915	4492	4882	4865	6775
L50	146	158	163	179	112
L75	345	372	355	392	264
# N's per 100 kbp	0	0	0	0	0

Assembly	RWJPA_16	RWJPA_17	RWJPA_18	RWJPA_19	RWJPA_20
Accession ID	JAIFHK000000000	JAMKOM000000000	JAMKON000000000	JAIFHJ000000000	JAIFHI000000000
# contigs (>= 0 bp)	673	1062	1203	958	966
# contigs (>= 1000 bp)	634	1062	1197	860	923
# contigs (>= 5000 bp)	382	140	198	373	360
# contigs (>= 10000 bp)	198	40	52	165	134
# contigs (>= 25000 bp)	48	7	7	21	22
# contigs (>= 50000 bp)	7	0	0	1	4
Total length (>= 0 bp)	6200164	3406309	4090970	5706942	5612846
Total length (>= 1000 bp)	6182263	3406309	4087789	5655797	5588878
Total length (>= 5000 bp)	5480934	1401816	1838827	4371530	4058536
Total length (>= 10000 bp)	4119577	717302	845068	2895414	2462214
Total length (>= 25000 bp)	1844529	249843	221521	733272	840069
Total length (>= 50000 bp)	463968	0	0	54103	252654
# contigs	650	1062	1201	910	948
Largest contig	85592	47909	43579	54103	86779
Total length	6192975	3406309	4090468	5690456	5607061
GC (%)	65.82	63.26	64.4	65.49	65.1
N50	15301	3992	4276	10113	8461
N75	8165	2202	2376	5438	4685
L50	114	208	243	161	172
L75	256	499	565	354	391
# N's per 100 kbp	0	0	0	0	0

Assembly	RWJPA_21	RWJPA_22	PA14	PAO1
Accession ID	JAIFHH000000000	JAIFHG000000000		
# contigs (>= 0 bp)	773	1095	841	1034
# contigs (>= 1000 bp)	744	1052	810	1020
# contigs (>= 5000 bp)	391	345	330	320
# contigs (>= 10000 bp)	172	114	140	104
# contigs (>= 25000 bp)	34	17	31	16
# contigs (>= 50000 bp)	5	1	3	3
Total length (>= 0 bp)	5803095	5441034	5531541	5167927
Total length (>= 1000 bp)	5788513	5423344	5516999	5161868
Total length (>= 5000 bp)	4857808	3592943	4223709	3406819
Total length (>= 10000 bp)	3298351	1995401	2872587	1905889
Total length (>= 25000 bp)	1286272	572392	1217268	629604
Total length (>= 50000 bp)	302790	60477	248864	158095
# contigs	757	1061	818	1025
Largest contig	69356	60477	97669	55294
Total length	5797699	5429815	5523386	5165173
GC (%)	65.56	65.05	65.51	65.6
N50	11604	7299	10834	7436
N75	6568	3881	5268	3804
L50	135	200	130	184
L75	302	454	315	428
# N's per 100 kbp	0	0	0	0
