# Supplementary material for: Pseudomonas aeruginosa clinical blood isolates display significant phenotypic variability
Source: PLoS One. 2022 Jul 6;17(7):e0270576. doi: 10.1371/journal.pone.0270576 (PMC9258867; doi:10.1371/journal.pone.0270576)
Supplement: S2 Table — (PDF) [file pone.0270576.s004.pdf]

S2 Table. Genes of Interest, their PAO1 reference name, and their associated biological activity category.

gene name	PAO1 reference number	biological activity category
alg44	PA3542_ref	Biofilm
algA	PA3551_ref	Biofilm
algB	PA5483_ref	Biofilm
algD	PA3540_ref	Biofilm
algE	PA3544_ref	Biofilm
algF	PA3550_ref	Biofilm
algG	PA3545_ref	Biofilm
algI	PA3548_ref	Biofilm
algJ	PA3549_ref	Biofilm
algK	PA3543_ref	Biofilm
algL	PA3547_ref	Biofilm
algR	PA5261_ref	Biofilm
algX	PA3546_ref	Biofilm
cdrA	PA4625_ref	Biofilm
flgB	PA1077_ref	Biofilm
flgC	PA1078_ref	Biofilm
flgE	PA1080_ref	Biofilm
flgF	PA1081_ref	Biofilm
flgG	PA1082_ref	Biofilm
flgH	PA1083_ref	Biofilm
flgI	PA1084_ref	Biofilm
flgK	PA1086_ref	Biofilm
flgL	PA1087_ref	Biofilm
flhA	PA1452_ref	Biofilm
flhB	PA1449_ref	Biofilm
fliC	PA1092_ref	Biofilm
fliF	PA1101_ref	Biofilm
fliG	PA1102_ref	Biofilm
fliM	PA1443_ref	Biofilm
fliN	PA1444_ref	Biofilm
fliO	PA1445_ref	Biofilm
fliP	PA1446_ref	Biofilm
fliQ	PA1447_ref	Biofilm
fliR	PA1448_ref	Biofilm
motA	PA4954_ref	Biofilm
motB	PA4953_ref	Biofilm

mucA	PA0763_ref	biofilm
pelA	PA3064_ref	Biofilm
pelB	PA3063_ref	Biofilm
pelC	PA3062_ref	Biofilm
pelD	PA3061_ref	Biofilm
pelE	PA3060_ref	Biofilm
pslA	PA2231_ref	Biofilm
pslB	PA2232_ref	Biofilm
pslC	PA2233_ref	Biofilm
pslD	PA2234_ref	Biofilm
pslE	PA2235_ref	Biofilm
pslF	PA2236_ref	Biofilm
pslG	PA2237_ref	Biofilm
pslH	PA2238_ref	Biofilm
pslI	PA2239_ref	Biofilm
pslJ	PA2240_ref	Biofilm
pslK	PA2241_ref	Biofilm
pslL	PA2242_ref	Biofilm
pslM	PA2243_ref	Biofilm
pslN	PA2244_ref	Biofilm
pslO	PA2245_ref	Biofilm
dnaE	PA3640_ref	Housekeeping
gacA	PA2586_ref	Housekeeping
gyrB	PA0004_ref	Housekeeping
mreB	PA4481_ref	Housekeeping
mutS	PA3620_ref	Housekeeping
ppsA	PA1770_ref	Housekeeping
recA	PA3617_ref	Housekeeping
rpoB	PA4270_ref	Housekeeping
rpoD	PA0576_ref	Housekeeping
algZ	PA3385_ref	Twitching
chpA	PA0413_ref	Twitching
pilA	PA4525_ref	Twitching
pilB	PA4526_ref	Twitching
pilE	PA4556_ref	Twitching
pilI	PA0410_ref	Twitching
pilJ	PA0411_ref	Twitching
pilR	PA4547_ref	Twitching
pilS	PA4546_ref	Twitching
pilT	PA0395_ref	Twitching

pilU	PA0396_ref	Twitching
phzA1	PA4210_ref	Pyocyanin Biosynthesis
phZA2	PA1899_ref	Pyocyanin Biosynthesis
phzB1	PA4211_ref	Pyocyanin Biosynthesis
phzB2	PA1900_ref	Pyocyanin Biosynthesis
phzC1	PA4212_ref	Pyocyanin Biosynthesis
phzC2	PA1901_ref	Pyocyanin Biosynthesis
phzD1	PA4213_ref	Pyocyanin Biosynthesis
phzD2	PA1902_ref	Pyocyanin Biosynthesis
phzE1	PA4214_ref	Pyocyanin Biosynthesis
phzE2	PA1903_ref	Pyocyanin Biosynthesis
phzF1	PA4215_ref	Pyocyanin Biosynthesis
phzF2	PA1904_ref	Pyocyanin Biosynthesis
phzG1	PA4216_ref	Pyocyanin Biosynthesis
phzG2	PA1905_ref	Pyocyanin Biosynthesis
phzM	PA4209_ref	Pyocyanin Biosynthesis
phzS	PA4217_ref	Pyocyanin Biosynthesis
FpvA	PA2398_ref	Pyocyanin Biosynthesis
FpvB	PA4168_ref	Pyocyanin Biosynthesis
PpyR	PA2663_ref	Pyocyanin Biosynthesis
PvdD	PA2399_ref	Pyocyanin Biosynthesis
PvdE	PA2397_ref	Pyocyanin Biosynthesis
PvdF	PA2396_ref	Pyocyanin Biosynthesis
gacS	PA0928_ref	Quorum Sensing
guaA	PA3769_ref	Quorum Sensing
lasI	PA1432_ref	Quorum Sensing
lasR	PA1430_ref	Quorum Sensing
phnA	PA1001_ref	Quorum Sensing
phnB	PA1002_ref	Quorum Sensing
pqsA	PA0996_ref	Quorum Sensing
pqsB	PA0997_ref	Quorum Sensing
pqsC	PA0998_ref	Quorum Sensing
pqsD	PA0999_ref	Quorum Sensing
pqsE	PA1000_ref	Quorum Sensing
pqsH	PA2587_ref	Quorum Sensing
pqsR (mvfR)	PA1003_ref	Quorum Sensing
rhlI	PA3476_ref	Quorum Sensing
rhlR	PA3477_ref	Quorum Sensing
rsaL	PA1431_ref	Quorum Sensing
vfr	PA0652_ref	Quorum Sensing
