# Supplementary material for: Pseudomonas aeruginosa clinical blood isolates display significant phenotypic variability
Source: PLoS One. 2022 Jul 6;17(7):e0270576. doi: 10.1371/journal.pone.0270576 (PMC9258867; doi:10.1371/journal.pone.0270576)
Supplement: S1 Fig — Genes involved in the (A) pyocyanin and pyoverdine, (B) biofilm, (C) twitching, and (D) quorum sensing were compared for similarity. (Left) Strains are ordered by their normalized phenotypic score from dark red indicating high to dark blue indicating low. (Right) Similarity scores between the gene in published PAO1 reference genome and ORF from our assembly. Dark purple on indicates 100 percent similarity with decreasing saturation indicating decreasing similarity. Grey boxes indicate that the gene was not found in the scaffolds of the strain and could indicate a loss of the gene or be the result of incomplete assembly of contigs. Checkerboard pattern indicates that the ORF was found running up to the edge of a contig. (PDF) [file pone.0270576.s001.pdf]

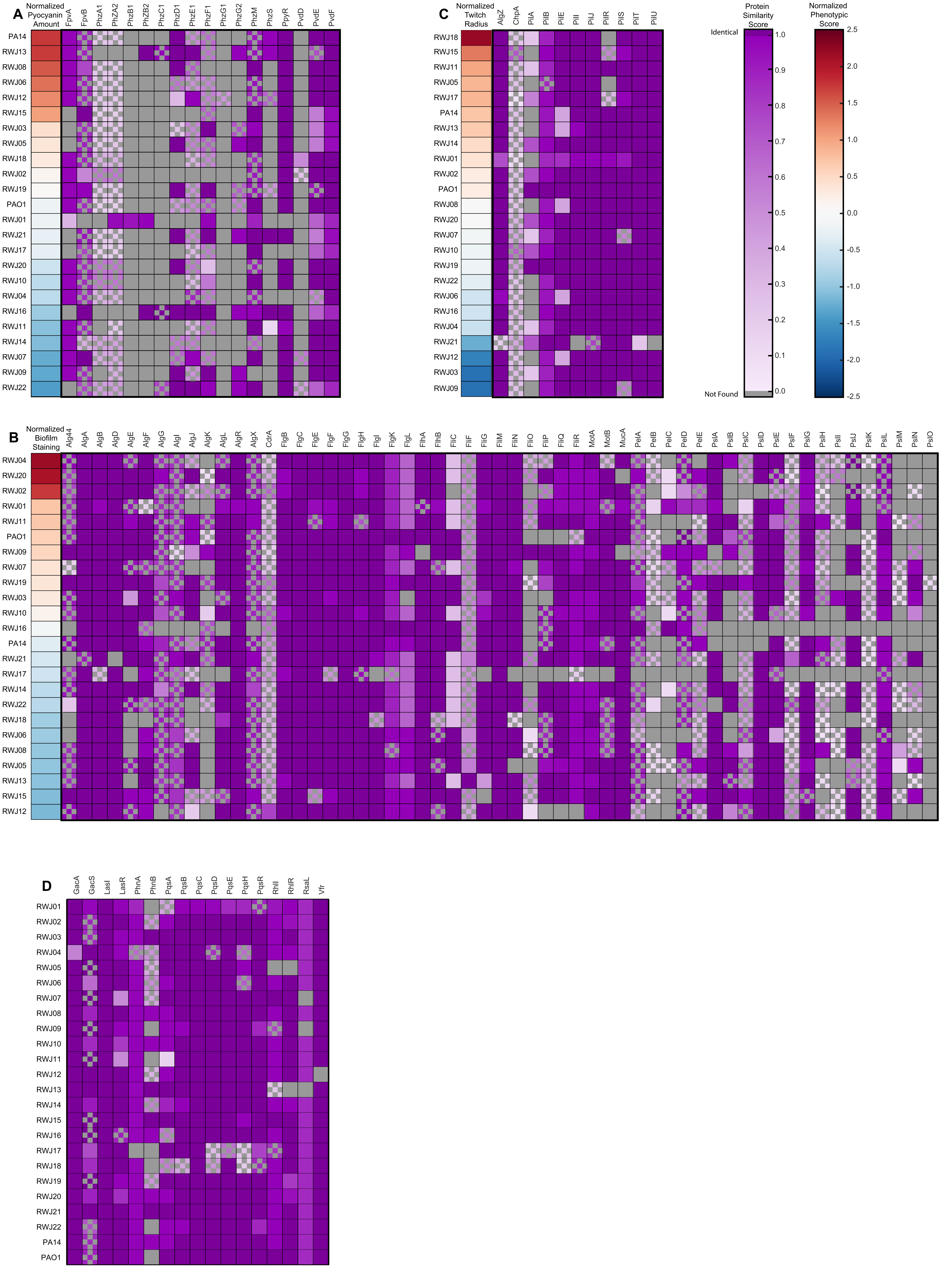
A
Normalized Pyocyanin Amount
FpvA
FpvB
PhzA1
PhzA2
PhzB1
PhzB2
PhzC1
PhzD1
PhzE1
PhzF1
PhzG1
PhzG2
PhzM
PhzS
PpyR
PvdD
PvdE
PvdF
PA14
RWJ13
RWJ08
RWJ06
RWJ12
RWJ15
RWJ03
RWJ05
RWJ18
RWJ02
RWJ19
PAO1
RWJ01
RWJ21
RWJ17
RWJ20
RWJ10
RWJ04
RWJ16
RWJ11
RWJ14
RWJ07
RWJ09
RWJ22
C
Normalized Twitch Radius
AlgZ
ChpA
PilA
PilB
PilE
PilI
PilJ
PilR
PilS
PilT
PilU
RWJ18
RWJ15
RWJ11
RWJ05
RWJ17
PA14
RWJ13
RWJ14
RWJ01
RWJ02
PAO1
RWJ08
RWJ20
RWJ07
RWJ10
RWJ19
RWJ22
RWJ06
RWJ16
RWJ04
RWJ21
RWJ12
RWJ03
RWJ09
Protein Similarity Score
Identical
Not Found
1.0
0.9
0.8
0.7
0.6
0.5
0.4
0.3
0.2
0.1
0.0
Normalized Phenotypic Score
2.5
2.0
1.5
1.0
0.5
0.0
-0.5
-1.0
-1.5
-2.0
-2.5
B
Normalized Biofilm Staining
Alg44
AlgA
AlgB
AlgD
AlgE
AlgF
AlgG
AlgI
AlgJ
AlgK
AlgL
AlgR
AlgX
CdrA
FlgB
FlgC
FlgE
FlgF
FlgG
FlgH
FlgI
FlgK
FlgL
FlhA
FlhB
FliC
FliF
FliG
FliM
FliN
FliO
FliP
FliQ
FliR
MotA
MotB
MucA
PelA
PelB
PelC
PelD
PelE
PslA
PslB
PslC
PslD
PslE
PslF
PslG
PslH
PslI
PslJ
PslK
PslL
PslM
PslN
PslO
RWJ04
RWJ20
RWJ02
RWJ01
RWJ11
PAO1
RWJ09
RWJ07
RWJ19
RWJ03
RWJ10
RWJ16
PA14
RWJ21
RWJ17
RWJ14
RWJ22
RWJ18
RWJ06
RWJ08
RWJ05
RWJ13
RWJ15
RWJ12
D
GacA
GacS
LasI
LasR
PhnA
PhnB
PqsA
PqsB
PqsC
PqsD
PqsE
PqsH
PqsR
RhlI
RhlR
RsaL
Vfr
RWJ01
RWJ02
RWJ03
RWJ04
RWJ05
RWJ06
RWJ07
RWJ08
RWJ09
RWJ10
RWJ11
RWJ12
RWJ13
RWJ14
RWJ15
RWJ16
RWJ17
RWJ18
RWJ19
RWJ20
RWJ21
RWJ22
PA14
PAO1
