# Supplementary material for: Pseudomonas aeruginosa clinical blood isolates display significant phenotypic variability
Source: PLoS One. 2022 Jul 6;17(7):e0270576. doi: 10.1371/journal.pone.0270576 (PMC9258867; doi:10.1371/journal.pone.0270576)
Supplement: S2 Fig — Genes involved in the quorum sensing were compared for similarity. Dark purple on indicates 100 percent similarity with decreasing saturation indicating decreasing similarity. Grey boxes indicate that the gene was not found in the scaffolds of the strain and could indicate a loss of the gene or be the result of incomplete assembly of contigs. Checkerboard pattern indicates that the ORF was found running up to the edge of a contig and was scored based on the portion of the protein present. (PDF) [file pone.0270576.s002.pdf]

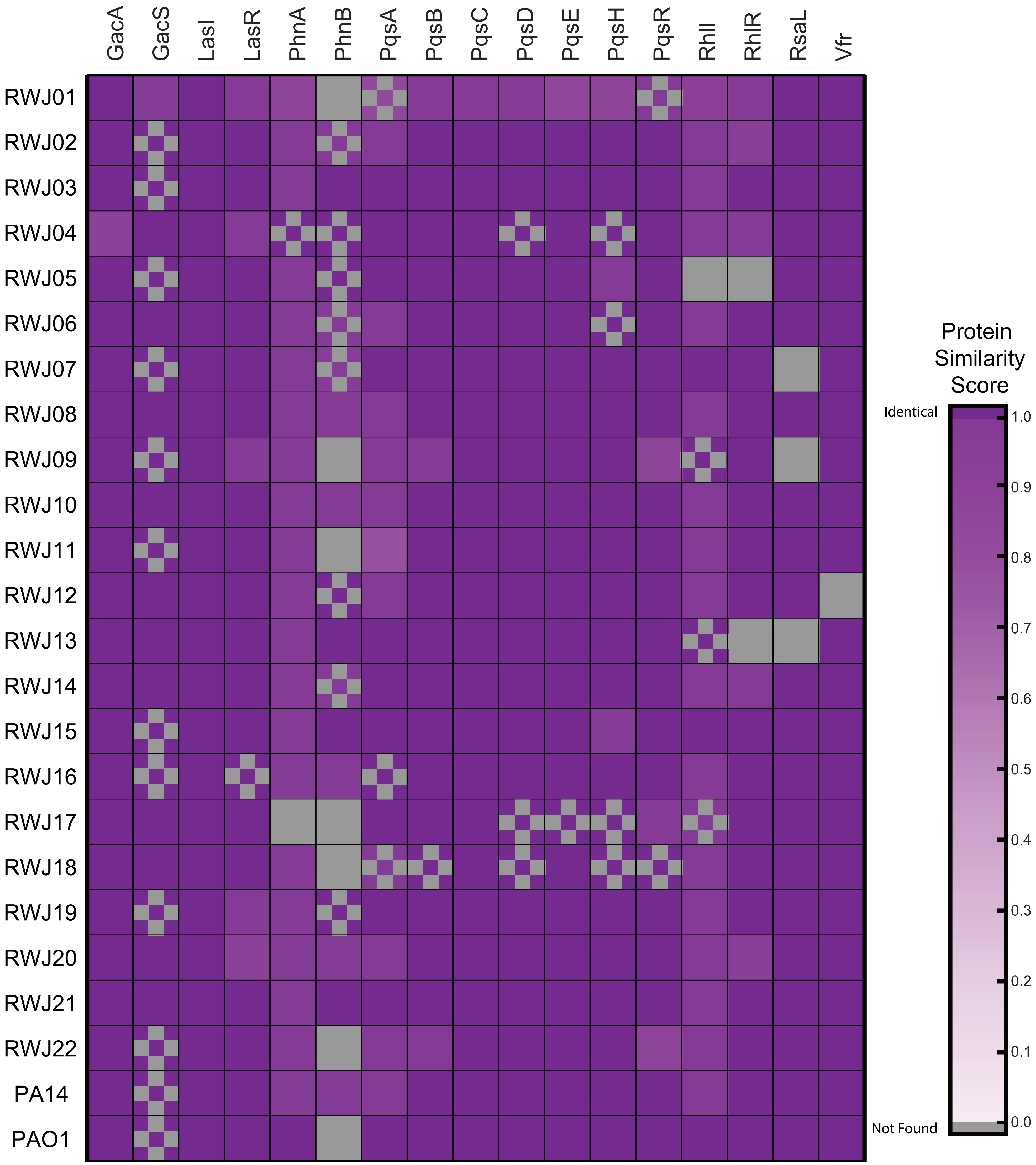
GacA
GacS
LasI
LasR
PhnA
PhnB
PqsA
PqsB
PqsC
PqsD
PqsE
PqsH
PqsR
RhlI
RhlR
RsaL
Vfr
RWJ01
RWJ02
RWJ03
RWJ04
RWJ05
RWJ06
RWJ07
RWJ08
RWJ09
RWJ10
RWJ11
RWJ12
RWJ13
RWJ14
RWJ15
RWJ16
RWJ17
RWJ18
RWJ19
RWJ20
RWJ21
RWJ22
PA14
PAO1
Protein Similarity Score
Identical
Not Found
1.0
0.9
0.8
0.7
0.6
0.5
0.4
0.3
0.2
0.1
0.0
